# Chronic low alcohol intake during pregnancy programs sex-specific cardiovascular deficits in rats

**DOI:** 10.1186/s13293-019-0235-9

**Published:** 2019-04-22

**Authors:** Sarah L. Walton, Melissa Tjongue, Marianne Tare, Edmund Kwok, Megan Probyn, Helena C. Parkington, John F. Bertram, Karen M. Moritz, Kate M. Denton

**Affiliations:** 1 Cardiovascular Disease Program, Monash Biomedicine Discovery Institute, Clayton, VIC Australia; 2 Department of Physiology, Monash University, Clayton, VIC Australia; 3 Monash Rural Health, Churchill, VIC Australia; 4 School of Biomedical Sciences, The University of Queensland, St Lucia, QLD Australia; 5 Department of Anatomy and Developmental Biology, Monash University, Clayton, VIC Australia; 6 Kidney Developmental Programming and Disease Laboratory, Monash Biomedicine Discovery Institute, Clayton, VIC Australia; 7 Child Health Research Centre, The University of Queensland, St Lucia, QLD Australia

**Keywords:** Fetal programming, Alcohol, Blood pressure, Vascular function

## Abstract

**Background:**

Exposure to an adverse environment in early life can have lifelong consequences for risk of cardiovascular disease. Maternal alcohol (ethanol) intake is common and associated with a variety of harmful effects to the fetus. However, examining the effects on the cardiovascular system in adult offspring has largely been neglected. The objectives of this study were to investigate the influence of chronic, low ethanol consumption throughout pregnancy on blood pressure, vascular reactivity and wall stiffness, all key determinants of cardiovascular health, in both male and female rat offspring.

**Methods:**

Female Sprague-Dawley rats were fed an ad libitum liquid diet ± 6% vol/vol ethanol throughout pregnancy. Male and female offspring were studied at 12 months of age. Arterial pressure, heart rate and locomotor activity were measured over 7 days via radiotelemetry. Renal lobar arteries were isolated and studied using wire and pressure myography.

**Results:**

Basal mean arterial pressure in female ethanol-exposed rats was reduced by ~ 5–6 mmHg compared to control female offspring, whereas arterial pressure was unaffected in male offspring. Ethanol-exposed offspring had an attenuated pressor response to an acute restraint stress, with this effect most evident in females. Renal artery function was not affected by prenatal ethanol exposure.

**Conclusions:**

We show for the first time that low level chronic maternal alcohol intake during pregnancy influences arterial pressure in adult offspring in the absence of fetal growth restriction.

**Electronic supplementary material:**

The online version of this article (10.1186/s13293-019-0235-9) contains supplementary material, which is available to authorized users.

## Background

Nearly 10% of women in the general population consume alcohol during pregnancy [1]. Alcohol is a known teratogen that readily crosses the placenta [2, 3], and consequently may directly exert adverse effects on developing organ systems of the fetus. Excessive levels of alcohol are associated with severe adverse outcomes during pregnancy, such as stillbirth [4], premature birth [5, 6] and intrauterine growth restriction [5]. In addition, overwhelming evidence shows high alcohol consumption during pregnancy is also associated with a set of life-long birth defects including craniofacial abnormalities, neurological deficits and fetal alcohol spectrum disorders (FASD) [7]. However, evidence concerning the impact on the cardiovascular system remains scant, particularly in response to what is perceived as moderate levels of alcohol intake. Children exposed to alcohol during gestation have higher rates of cardiovascular malformations [8], kidney defects [9, 10] and evidence of vascular stiffening [11]. It is unknown whether these deficits persist into adulthood. Since the developing kidney and vasculature, pivotal in the regulation of arterial pressure throughout life, appear particularly susceptible to in utero insults [12–15], it is plausible that repeated alcohol exposure during development may trigger increased risk of cardiovascular dysfunction in adult offspring.

Experimental models of fetal alcohol exposure have identified changes in the heart, small arteries and kidneys that may confer increased cardiovascular risk. In fetal sheep, repeated maternal ethanol (EtOH) administration (a ‘binge’ model; 0.75 g EtOH/kg) during late gestation, resulting in a fetal blood-alcohol concentration (BAC) of ~ 0.12%, was associated with marked arterial stiffening and alterations in vasodilator function that were dependent upon the vascular bed studied [16] and a reduction in nephron number per kidney [17]. Furthermore, in a rat binge model of EtOH consumption during mid-gestation (1 g EtOH/kg, embryonic day [E] 13.5-E14.5), where the maternal BAC reached ~ 0.11%, nephron number was reduced and mean arterial pressure increased in both sexes at 6 months of age [18]. Chronic exposure to ethanol throughout pregnancy (6.36% vol/vol EtOH in liquid diet, E2-birth) led to elevated mean arterial pressure (MAP) and impaired aortic endothelium-dependent relaxation in rat offspring at 25 weeks of age [19]. A similar study in rats (6% vol/vol EtOH in liquid diet, E0 to birth) showed evidence of left ventricular hypertrophy and cardiac fibrosis male and female offspring at 8 months of age [20]. These results indicate that excessive and/or prolonged ethanol exposure during gestation has long-lasting effects on cardiovascular health.

Our laboratory has established a rat model of low exposure to EtOH throughout pregnancy. Pregnant rats are fed a liquid diet containing 6% vol/vol EtOH throughout the entirety of gestation, resulting in a peak blood-alcohol concentration of ~ 0.03% [21]. We have previously reported the feeding regime and consumption pattern in detail [21]. In brief, the diet was offered fresh daily at commencement of the dark (active) cycle and was available over most of the day (21 h). The blood-alcohol concentration was measured in a subset of animals 30 min and 5 h after offering the fresh diet. At 30 min, the BAC averaged ~ 0.03% and by 5 h was undetectable in most animals. This model of maternal EtOH exposure does not affect maternal nutrition or pregnancy outcomes, such as litter size or birth weight [22], and the level of alcohol exposure is equivalent to approximately two standard drinks within an hour in humans. We hypothesised that chronic low prenatal EtOH exposure would alter blood pressure profiles, endothelial vasodilator function and arterial stiffness in the offspring at 12 months of age, equivalent to middle age in humans. To address this hypothesis, arterial pressures were measured via radiotelemetry in male and female offspring, given known sex-differences in cardiovascular risk [23, 24]. The pressor (rise in blood pressure) and tachycardiac responses to restraint stress were assessed as indices of cardiovascular reactivity, a predictor of future blood pressure status [25, 26]. Endothelial and smooth muscle function, nerve-mediated constriction and arterial wall stiffness were assessed in isolated renal lobar arteries; when disrupted, these variables may contribute to adverse cardiovascular sequelae. We focussed on renal arteries as we have previously shown the reactivity and passive wall properties in these arteries to be significantly influenced in a sheep model of maternal alcohol exposure [16]. The offspring were examined at 12 months of age as loss of cardiovascular protection has previously been observed at this age in female rats in other models of fetal programming of cardiovascular disease [27].

## Methods

### Animals

Experiments were conducted in accordance with the Australian Code of Practice for the Care and Use of Animals for Scientific Purposes and approved by both the University of Queensland Animal Ethics Committee and the Monash University School of Biomedical Sciences Animal Ethics Committee. Animals were obtained from the Animal Resources Centre (Perth, WA, Australia) and housed in an experimental room with temperature maintained at 25 °C and a 12-h light-dark cycle, and maintained on standard rat chow and water, unless otherwise stated. Rats were allowed 1–2 weeks to acclimatise prior to the commencement of this study protocol.

Female nulliparous Sprague-Dawley rats of 8 weeks of age and ~ 280 g were mated overnight with untreated males. Pregnancy was confirmed by the presence of seminal plugs and recorded as embryonic (E) day 1. Pregnant rats were then randomly allocated to receive a liquid diet containing 6% vol/vol EtOH (EtOH-exposed: *n* = 8 dams) or an isocaloric diet (control: *n* = 9 dams) ad libitum throughout the entirety of pregnancy, as previously described [28]. At parturition, the liquid diets were removed and the dams were provided ad libitum access to standard rat chow and water. Offspring were weaned at postnatal (PN) day 30. Blood pressure and vascular function and structure were assessed in male and female offspring at 12 months of age. Only one offspring per sex per litter was included in any group.

### Blood pressure

At 12 months of age, rats were anaesthetized (isoflurane; 2–5% *v*/*v* O_2_) for implantation of a radiotelemetry probe (PA-C40, Data Sciences International, MN, USA) into the abdominal aorta, as described previously [29]. Following a 10-day recovery period, systolic blood pressure (SBP), diastolic blood pressure (DBP), mean arterial pressure (MAP), pulse pressure (PP), heart rate (HR) and locomotor activity were determined over 7 days, with sampling for 10 s every 10 min, using a Dataquest ART data acquisition system (Data Sciences International, MN, USA).

### Response to restraint stress

On the eighth day of measurement, rats were subjected to a restraint stress challenge during the light period. Data were acquired every 10 s for 30 min to establish a baseline. Each rat was then guided into a cylindrical plexiglass restrainer to confine the animal for 30 min, then released into the home cage.

### Assessment of vascular function

At the conclusion of the in vivo studies, rats were anaesthetised and renal lobar arteries were dissected in physiological salt solution (PSS) for vascular function determination. Rings of renal lobar artery (each 1–2 mm in length) were mounted on a four-channel wire myograph (Model 610 M, Danish Myo Technology, Aarhus, Denmark) and bathed in PSS bubbled with carbogen (95% O_2_, 5% CO_2_ at 36 °C) to test vascular reactivity, as previously described [30]. The integrity of the endothelium was confirmed, as demonstrated by complete relaxation following stimulation for acetylcholine (ACh, 10^−5^ M) in arteries submaximally constricted with phenylephrine (PE).

To test smooth muscle contraction, arterial rings were exposed to cumulative concentrations of PE (10^−9^–10^−4^ M) and angiotensin II (AngII, 10^−10^–10^−5^ M). Contractions were expressed as a percentage of the contraction evoked by HiK. Endothelium-dependent relaxation was tested in submaximally preconstricted (~ 70% of maximal) arteries using cumulative application of acetylcholine (ACh; 10^−9^–10^−6^ M). Responses were obtained before and after sequential blockade of nitric oxide synthase (NOS) with N(ω)-nitro-l-arginine methyl ester (L-NAME, 2 × 10^−4^ M) and cyclooxygenase inhibitor indomethacin (INDO; 10^−6^ M). Relaxation remaining in the presence of L-NAME and INDO was attributed to the actions of endothelium-derived hyperpolarisation (EDH). In submaximally preconstricted arteries, cumulative addition of the nitric oxide (NO) donor, sodium nitroprusside (SNP; 10^−9^–10^−5^ M), was used to test endothelium-independent relaxation.

### Response of the renal artery to perivascular nerve stimulation

For perivascular nerve stimulation, renal lobar arteries were mounted onto a single channel wire myograph (Monash University, Melbourne, Australia) and continuously superfused with PSS at 36 °C and bubbled with carbogen. Endothelial viability was tested for each artery, as described above. Platinum electrodes, positioned on either side of the artery, were used to stimulate the perivascular nerves, as previously described [31, 32]. Arteries were stimulated transmurally using a Grass S88 stimulator (Quincy, Massachusetts, USA). The stimulus consisted of continuous trains of pulses (each 0.1 ms in duration) at 1–8 Hz, applied for 5 s, at increasing intensities (50–150 V, dial settings). Nerve stimulation was then repeated following blockade of α_1_-adrenoreceptors with prazosin (10^−6^ M), and tetrodotoxin (10^−7^ M) to confirm that the recorded constrictions were reflective of nerve stimulation rather than direct smooth muscle stimulation. All responses to perivascular nerve stimulation were expressed as a percentage of contraction evoked by HiK PSS.

### Testing passive wall properties

Passive mechanical wall properties were determined in leak-free segments of renal arteries (3–5 mm in length, ~ 400 μm outside diameter) mounted onto a pressure myograph (Danish Myo Technology, Denmark) and superfused at 15 ml/min with zero-Ca^2+^ PSS containing 2 mM EGTA at 36 °C, as previously described [30]. Intraluminal pressure was increased from 5 to 110 mmHg in 10 mmHg increments. Wall thickness and outside diameter at each pressure were measured and used to calculate wall stress and wall strain [30, 33].

### Quantitative real-time PCR

RNA was extracted from renal arteries using Qiazol (Qiagen, Chadstone Centre, VIC, Australia). All RNA was treated with deoxyribonuclease I and assessed for purity (260/280 and 260/230 ratios) and yield using a NanoDrop (Thermo Fischer) spectrophotometer. One microgram of RNA was reverse transcribed into cDNA (iScript, BioRad, Gladesville, NSW, Australia) in a 10 μl reaction volume containing 25 ng of cDNA and 10 pM of each primer. All assays were performed in duplicate. PCR primer sequences are provided in Additional File 1: Table S1. The comparative cycle threshold method was used for all expression assays using the mean of *18s* and *Hprt* as the endogenous control. mRNA levels were normalised to the mean of the control male group.

## Statistical analysis

Data are expressed as mean ± SEM, where *n* represents the number of animals. Telemetry data were analysed using a two-way repeated measures ANOVA, with EtOH treatment (*P*_trt_) and time (*P*_time_) or sex (*P*_sex_) as factors. In addition, 24-h time series telemetry data were analysed using the cosinor analysis to produce estimates of 24-h periodicity (mesor, amplitude and acrophase; Matlab, Version 9.2.0, The MathWorks Inc., Natick, Massachusetts, USA). Concentration-response data to vasoconstrictors and vasodilators were fitted with sigmoidal curves using the least-squares method (GraphPad Prism, San Diego, CA, USA). For the ACh curves, curves were fitted to data from baseline to the maximal response (10^−6^ M ACh). From these curves, sensitivity (negative logarithm of the effective concentration for half-maximal response, pEC_50_) and maximal response (*R*_max_) were determined and analysed using two-way repeated measures ANOVA with EtOH treatment and sex as factors. Responses to perivascular nerve stimulation were assessed using repeated measures ANOVA with the factors of EtOH treatment and frequency/voltage. Stress-strain relationships were analysed using a repeated measures two-way ANOVA with the factors treatment and strain. Sidak post-hoc tests were performed where appropriate. *P* ≤ 0.05 was accepted as statistically significant.

## Results

### Reduced arterial pressure in EtOH-exposed female offspring

**Fig. 1:**
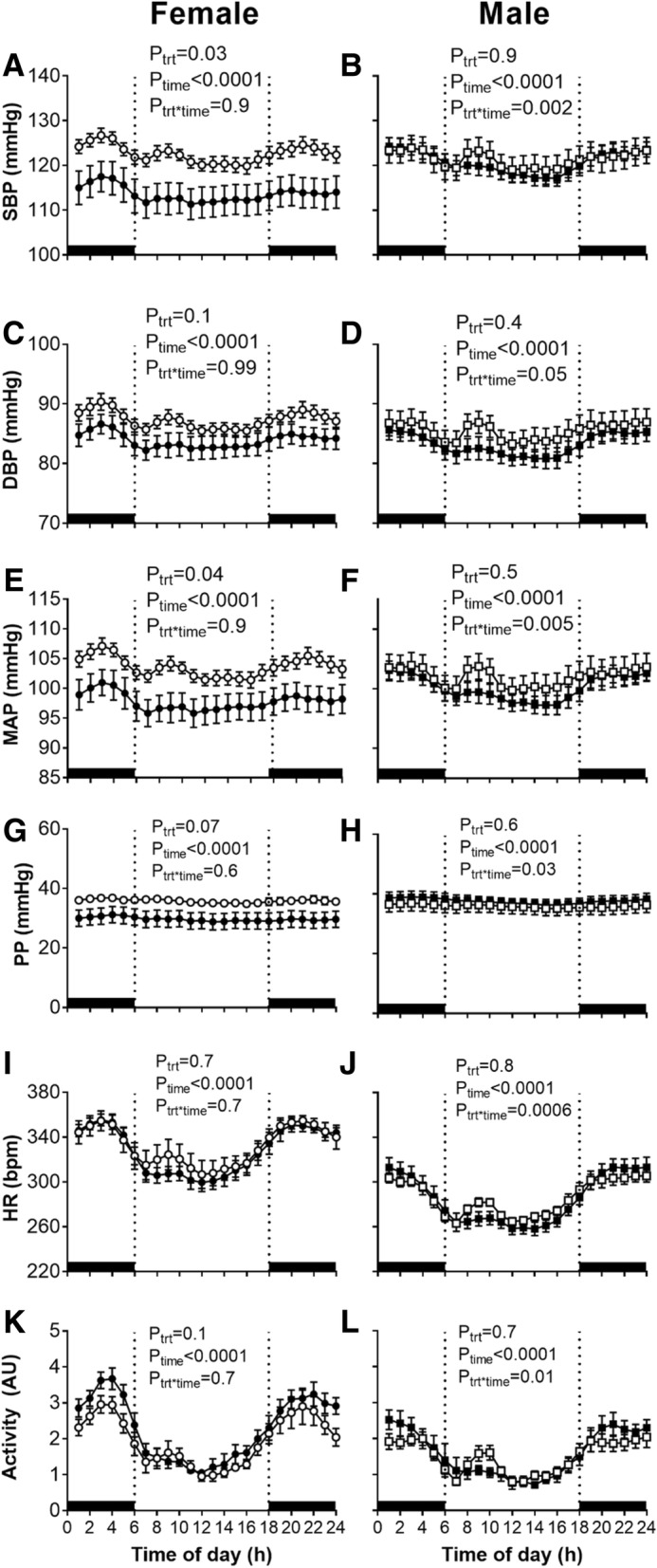
Arterial pressure, heart rate and locomotor activity over 24 h in 12-month-old offspring. Systolic blood pressure (SBP), diastolic blood pressure (DBP), mean arterial pressure (MAP), pulse pressure (PP), heart rate (HR) and locomotor activity over 24 h in female (left) and male (right) offspring. Control: open points; EtOH-exposed: closed points. Values are mean ± SEM, *n* = 7–9/group. Data analysed using a two-way repeated measures ANOVA with the factors treatment, time and their interaction. Sidak post-hoc tests were performed where appropriate

Systolic blood pressure was significantly lower in EtOH-exposed females over the light period (control: 121 ± 1 mmHg; EtOH-exposed: 112 ± 3 mmHg) and over the dark period (control: 124 ± 2 mmHg; EtOH-exposed: 115 ± 4 mmHg; Fig. 1a, *P*_trt_ = 0.03, *P*_time_ < 0.0001) with respect to control females. This was reflected by a decrease in MAP of ~ 5–6 mmHg in EtOH-exposed females compared with control females over the 24-h period (Fig. 1e). DBP (Fig. 1c), PP (Fig. 1g), HR (Fig. 1i) and locomotor activity (Fig. 1k) were not affected by prenatal EtOH exposure in female offspring. In male offspring, there were no consistent differences in arterial pressure, HR or locomotor activity over the 24-h period (Fig. 1; all *P*_trt_ > 0.05). However, two-way ANOVA of arterial pressure, HR and locomotor activity revealed an interaction between EtOH exposure and time (Fig 1; all *P*_trt*time_ < 0.01). This was attributed to a spike in HR and locomotor activity observed in control male offspring between 08:00 to 10:00 h in the light period, at which point the room was entered by staff for husbandry purposes. In contrast, EtOH-exposed males did not exhibit the spike in HR and locomotor activity observed in control males. HR and locomotor activity were significantly greater in female offspring compared with males, irrespective of EtOH exposure (Table 1; *P*_sex_ < 0.01).

**Table 1:** Mesor, amplitude and acrophase of MAP, heart rate and locomotor activity at 12 months of age over a 24-h cycle time (CT) following cosinor analyses

	Female		Male		Two-way ANOVA		
	Control	EtOH-exposed	Control	EtOH-exposed	*P*_trt_	*P*_sex_	*P*_trt*sex_
SBP							
Mesor (mmHg)	123 ± 1	114 ± 3*	121 ± 3	121 ± 2	*P* = 0.07	NS	NS
Amplitude (mmHg)	2.6 ± 0.6	2.3 ± 0.4	2.8 ± 0.3	3.4 ± 0.4	NS	NS	NS
Acrophase, CT (h)	3.6 ± 1.0	5.8 ± 2.1	5.0 ± 1.4	2.9 ± 0.5	NS	NS	NS
DBP							
Mesor (mmHg)	87 ± 1	84 ± 2	85 ± 2	83 ± 2	NS	NS	NS
Amplitude (mmHg)	2.0 ± 0.5	1.8 ± 0.2	2.1 ± 0.3	2.4 ± 0.4	NS	NS	NS
Acrophase, CT (h)	2.8 ± 1.2	4.4 ± 2.4	4.2 ± 1.6	2.1 ± 0.4	NS	NS	NS
MAP							
Mesor (mmHg)	104 ± 1	98 ± 2	102 ± 2	100 ± 1	*P* = 0.05	NS	NS
Amplitude (mmHg)	2.2 ± 0.5	2.0 ± 0.3	2.4 ± 0.3	2.8 ± 0.4	NS	NS	NS
Acrophase, CT (h)	3.2 ± 1.0	5.1 ± 2.2	4.7 ± 1.5	2.4 ± 0.5	NS	NS	NS
HR							
Mesor (bpm)	332 ± 9	328 ± 6	286 ± 3	285 ± 6	NS	*P* < 0.0001	NS
Amplitude (bpm)	24 ± 4	28 ± 3	21 ± 3	31 ± 3^†^	*P* = 0.04	NS	NS
Acrophase, CT (h)	2.2 ± 0.7	1.6 ± 0.3	1.7 ± 0.6	1.4 ± 0.5	NS	NS	NS
Activity							
Mesor (AU)	2.0 ± 0.1	2.3 ± 0.1	1.5 ± 0.1	1.6 ± 0.2	NS	*P* = 0.0007	NS
Amplitude (AU)	1.0 ± 0.2	1.2 ± 0.1	0.6 ± 0.1	0.9 ± 0.1	*P* = 0.06	*P* = 0.01	NS
Acrophase, CT (h)	3.0 ± 1.0	2.1 ± 0.4	2.7 ± 1.0	1.9 ± 0.5	NS	NS	NS

*P < 0.05 comparing control and EtOH-exposed female offspring and ^†^*P* = 0.07 comparing control/EtOH-exposed male offspring, from Sidak’s multiple comparisons tests

We explored the circadian regulation of arterial pressure, HR and locomotor activity further by performing cosinor analysis (Fig. 1, all *P*_time_ < 0.001; Table 1), with all values being greater during the dark period (18:00 h to 06:00 h) compared with the light period (06:00 h to 18:00 h). Cosinor analysis revealed an overall effect of prenatal EtOH exposure on increased wave amplitude of HR over a 24-h period (Table 1; *P*_trt_ = 0.04), with this effect most evident in EtOH-exposed male offspring (*P* = 0.07 vs. control males).

### Attenuated pressor response to restraint stress in EtOH-exposed male and female offspring

**Fig. 2:**
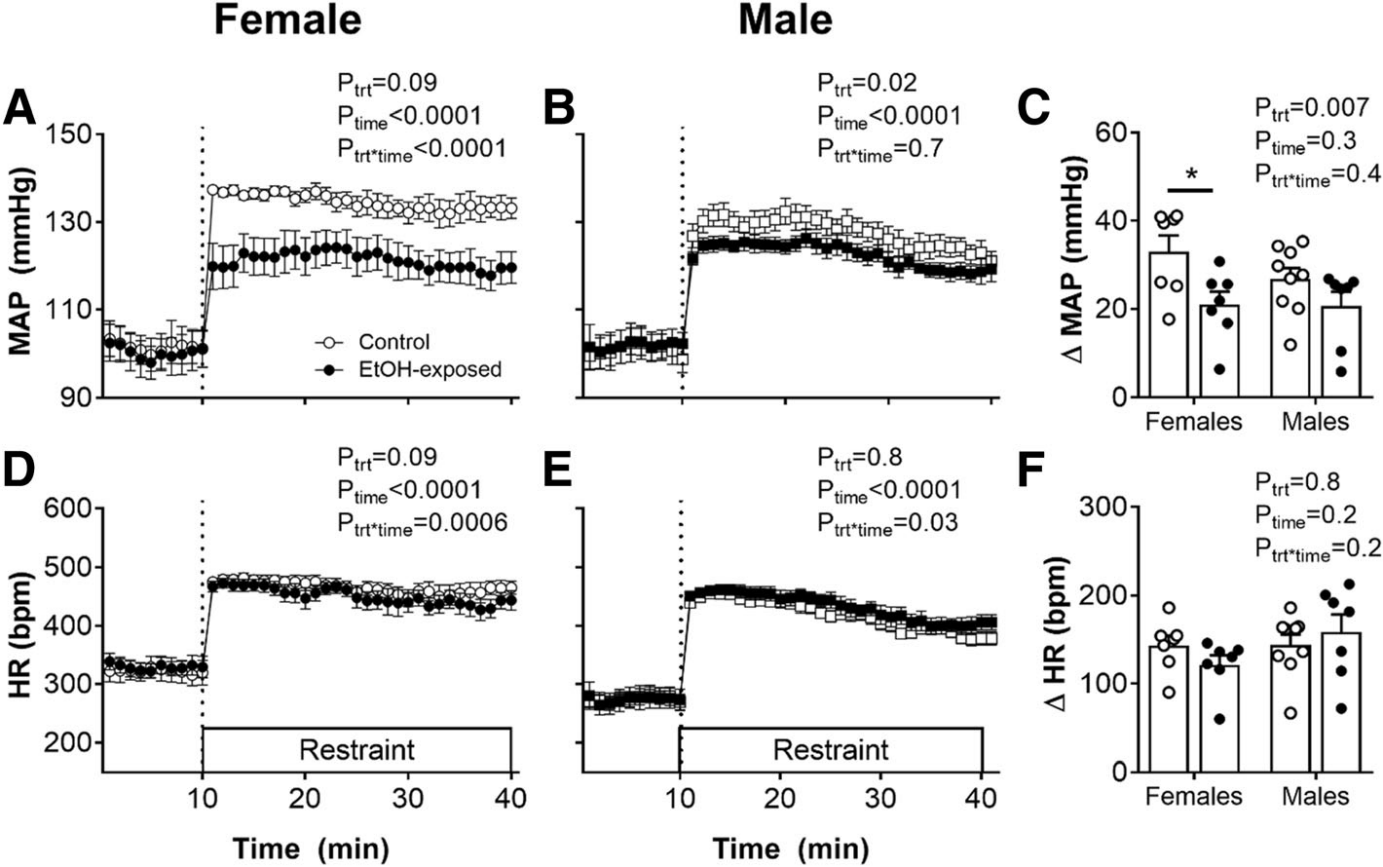
Mean arterial pressure and heart rate responses to acute restraint stress in 12-month-old offspring. Mean arterial pressure (MAP) before and during 30 min of restraint stress in female (**a**) and male (**b**) offspring, and change (Δ) in MAP from baseline to the average of 30 min of restraint stress (**c**). Heart rate (HR) before and during restraint stress in female (**d**) and male (**e**) offspring, and ΔHR from baseline to the average of 30 min of restraint stress (**f**). Control: open bars/points; EtOH-exposed: closed bars/points. Values are mean ± SEM, *n* = 7–9/group. Data analysed using a two-way ANOVA with the factors treatment, sex and their interaction. *P < 0.05 from a Sidak post-hoc test

Restraint stress induced immediate and sustained pressor and tachycardiac responses in both control and EtOH-exposed rats (Fig. 2; *P*_time_ < 0.0001). However, the pressor response over 30 min of restraint was reduced in EtOH-exposed offspring, compared with control counterparts (Fig. 2c; *P*_trt_ = 0.007). Post-hoc analysis revealed that this effect was greatest in female EtOH-exposed offspring, compared with control females (Fig. 2c; *P* < 0.05). The tachycardiac response was similar in control and EtOH-exposed female (Fig. 2d) and male (Fig. 2e) offspring.

### Preservation of renal lobar artery reactivity

**Fig. 3:**
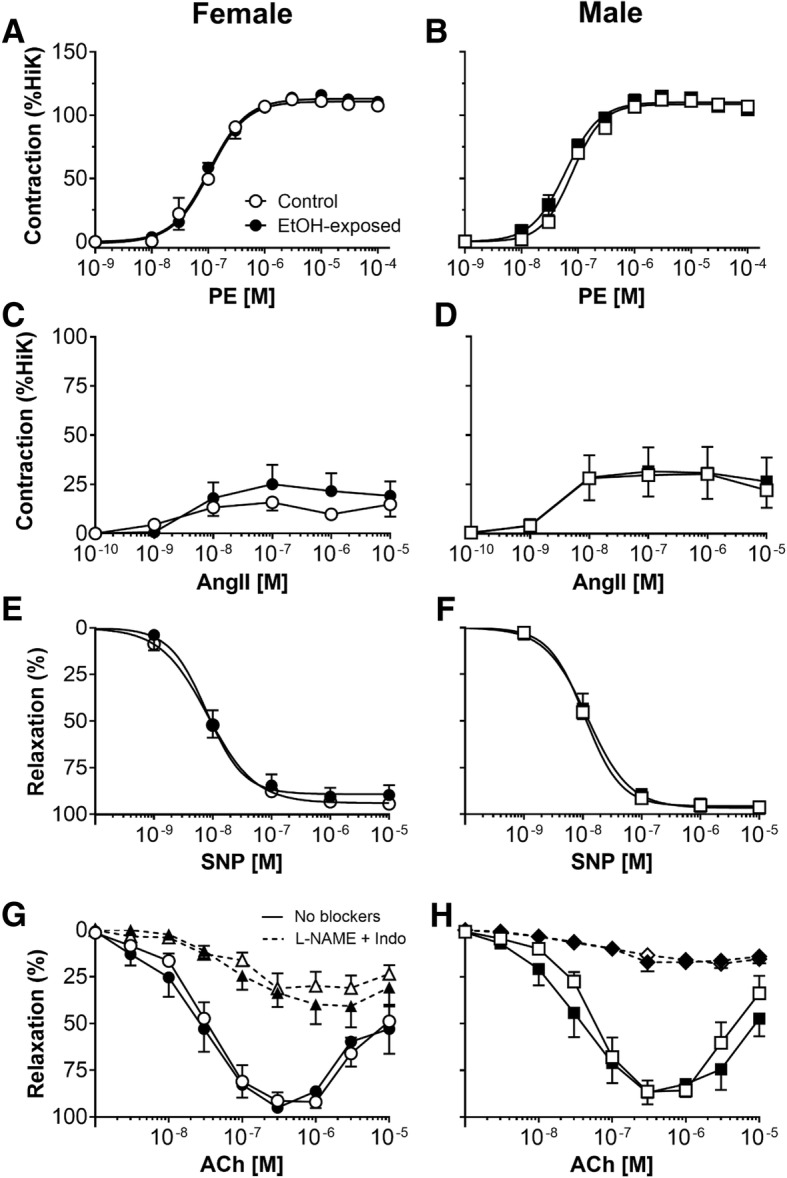
Smooth muscle contraction and relaxation, and endothelium-dependent relaxation of renal lobar arteries. Renal arteries were obtained from 12-month-old female (left) and male (right) offspring. Functional responses to **a**, **b** phenylephrine (PE), **c, d** angiotensin II (AngII), **e**, **f** sodium nitroprusside (SNP) and **g**, and **h** acetylcholine (ACh) either in the presence or absence of the blockers L-NAME + Indo. Control: open points; EtOH-exposed: closed points. Values are mean ± SEM, *n* = 6–8/group

There was no difference in the sensitivity or maximal contraction of the renal arteries to the α_1_-adrenoreceptor agonist phenylephrine between control and EtOH-exposed offspring of both sexes (Fig. 3a, b; Table 2). AngII evoked concentration-dependent contraction, and the sensitivity and maximal responses were not significantly different between control and EtOH offspring or between the sexes (Fig. 3c, d; Table 2). SNP elicited concentration-dependent relaxation in renal arteries, and neither maximal relaxation nor the pEC50 were different between control and EtOH-exposed offspring of both sexes (Fig. 3e, f; Table 2).

**Table 2:** Renal lobar artery reactivity

	Females		Males		Two-way ANOVA		
	Control	EtOH-exposed	Control	EtOH-exposed	*P*_trt_	*P*_sex_	*P*_trt*sex_
HiK							
Contraction (mN mm^−1^)	10.9 ± 0.4	11.4 ± 0.7	11.4 ± 1.1	10.8 ± 0.8	NS	NS	NS
PE							
pEC_50_	7.03 ± 0.15	6.96 ± 0.12	7.12 ± 0.04	7.25 ± 0.06	NS	NS	NS
Max. contraction (% HiK)	113 ± 2	117 ± 2	111 ± 3	116 ± 4	NS	NS	NS
AngII							
pEC_50_	8.17 ± 0.26	8.16 ± 0.09	8.47 ± 0.15	8.44 ± 0.17	NS	NS	NS
Max. contraction (% HiK)	21 ± 6	26 ± 10	33 ± 13	34 ± 13	NS	NS	NS
SNP							
pEC_50_	8.07 ± 0.10	8.05 ± 0.07	7.95 ± 0.10	7.89 ± 0.09	NS	NS	NS
*R*_max_ (%)	95 ± 2	91 ± 5	96 ± 3	97 ± 1	NS	NS	NS
ACh							
pEC_50_	6.85 ± 0.23	6.99 ± 0.15	6.79 ± 0.09	6.97 ± 0.18	NS	NS	NS
*R*_max_ (%)	94 ± 3	97 ± 1	89 ± 6	92 ± 6	NS	NS	NS
ACh + L-NAME + INDO							
pEC_50_	7.18 ± 0.21	6.98 ± 0.24	7.27 ± 0.16	7.26 ± 0.32	NS	NS	NS
*R*_max_ (%)	34 ± 7	48 ± 10	19 ± 3	21 ± 5	NS	*P* = 0.04	NS

Values are mean ± SEM (*n* = 6–7 per group). The effect of prenatal treatment (trt), sex or their interaction (trt*sex) was evaluated by two-way ANOVA. *Max*. maximal

Stimulation of the endothelium with ACh (up to 10^−6^ M) evoked concentration-dependent relaxation in the renal arteries (Fig. 3g, h). At higher concentrations of ACh (> 10^−6^ M), relaxation amplitude was reduced in all treatment groups. Neither the sensitivity nor the maximal relaxation evoked by ACh was different between treatment groups or sexes (Table 2). The relaxation attributed to EDH was revealed in the presence of L-NAME and INDO. Maximal EDH-mediated relaxation was similar between renal arteries of control and EtOH-exposed offspring (Fig. 3g, h; Table 2). However, maximal EDH-mediated relaxation was larger in renal arteries from female compared with male offspring (*P*_sex_ = 0.04; Table 2). There was no difference between the treatment groups or sexes in the ability of renal arteries to contract to HiK (Table 2).

### Neurovascular constriction

**Fig. 4:**
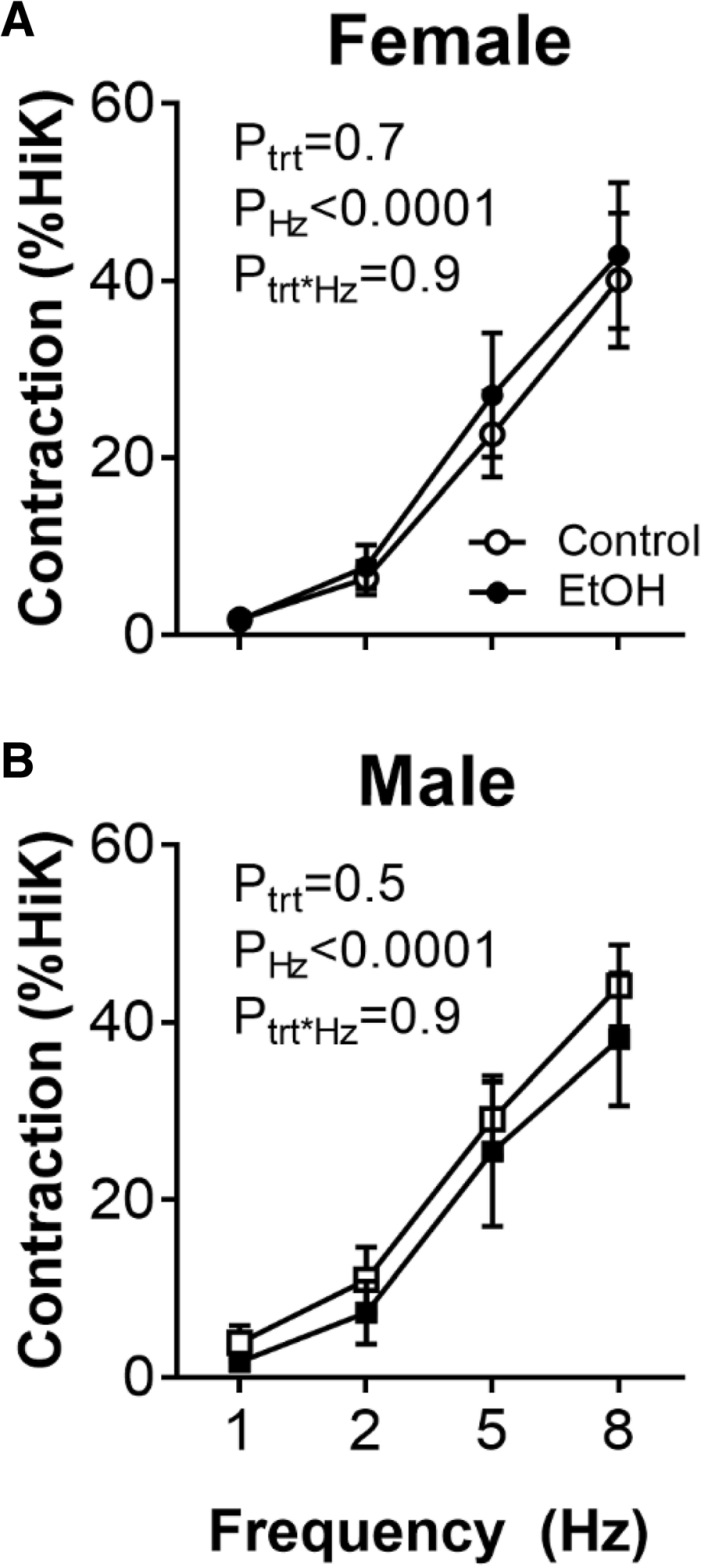
Neurovascular constriction in isolated renal lobar arteries from offspring at 12 months of age. Contraction evoked by perivascular nerve stimulation, 5-s trains of pulses of increasing frequency at 150 V (dial setting), in renal lobar arteries from **a** female and **b** male offspring. Control: open points; EtOH-exposed: closed points. Values are mean ± SEM, *n* = 6–8/group. Data analysed using a repeated measures two-way ANOVA with the factors treatment, frequency (Hz) and their interaction. Sidak post hoc tests were performed where appropriate

Perivascular nerve stimulation of increasing frequency evoked contractions of increasing amplitude (*P*_Hz_ < 0.0001; Fig. 4), with no differences between treatment groups. Single pulses delivered at increasing stimulus strength evoked contractions of increasing amplitude in control female offspring (*P*_voltage_ < 0.0001; Additional File 2). However, these neurovascular constrictions were attenuated in arteries of female EtOH-exposed females compared with control females (*P*_trt_ = 0.02; Additional File 2). No significant differences were observed between male treatment groups (Additional File 2). Neurovascular constrictions were markedly attenuated by the α_1_-adrenoreceptor blocker, prazosin and the remaining responses were completely abolished in the presence of tetrodotoxin in all groups (Additional File 2), thus confirming neurogenicity of responses.

### Arterial passive mechanical wall properties

**Fig. 5:**
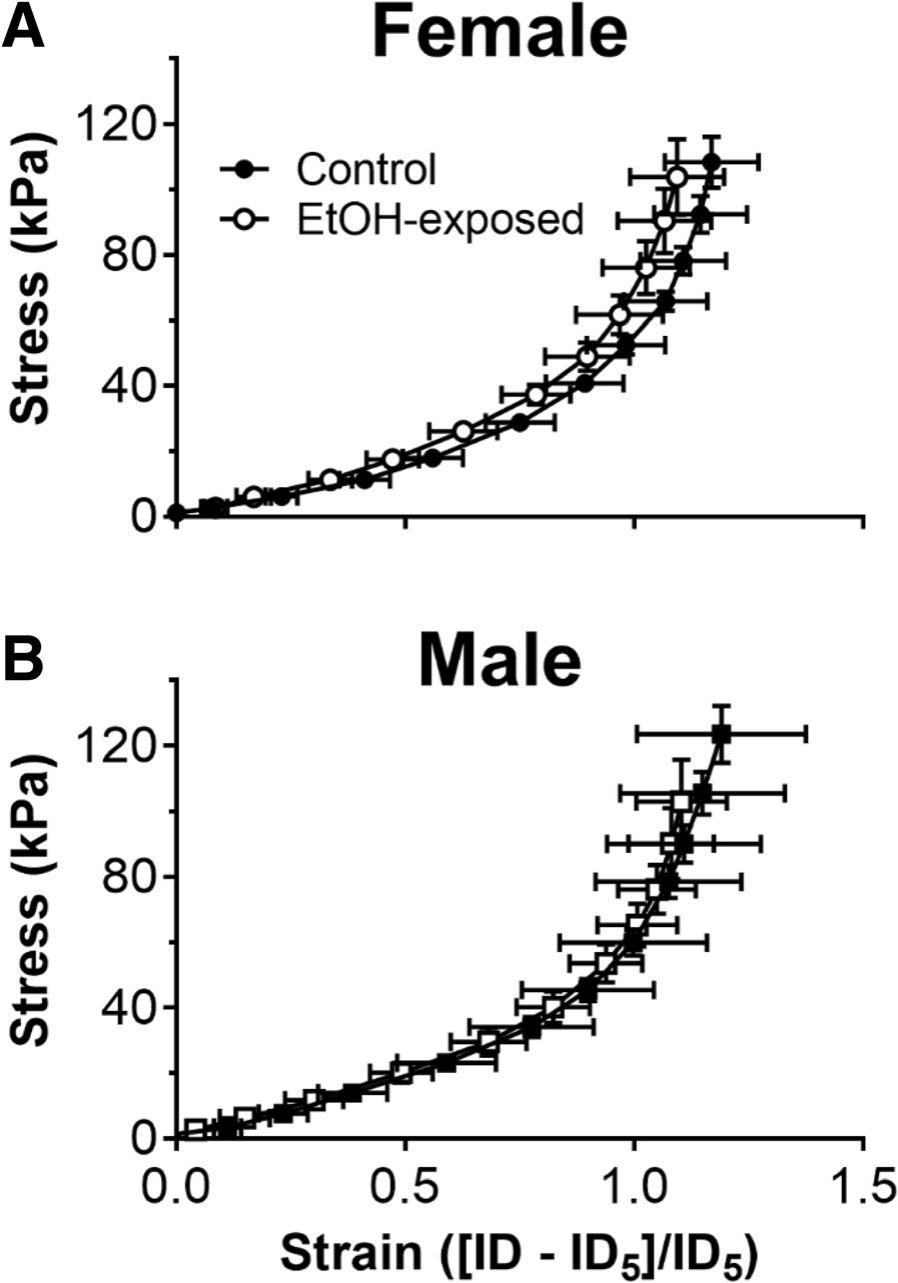
Passive mechanical wall properties in renal lobar arteries at 12 months of age. Passive stress-strain relationships for renal arteries from **a** female and **b** male offspring. Control: open points; EtOH-exposed: closed points. Values are mean ± SEM, *n* = 6–8/group. Data analysed using a repeated-measures two-way ANOVA

The outside diameters of the renal lobar arteries were not different between treatment groups. However, outside diameter of renal lobar arteries of male offspring was larger compared with those of arteries from females (control female: 379 ± 13 μm, EtOH female: 390 ± 13 μm, control male: 447 ± 27 μm, EtOH male: 397 ± 27 μm; *P*_trt_ = 0.3, *P*_sex_ = 0.04). There were no differences in the stress-strain relationships for renal arteries between control and EtOH-exposed females (Fig. 5a) and males (Fig. 5b).

### Quantitative real-time PCR analysis of gene expression in renal arteries

**Fig. 6:**
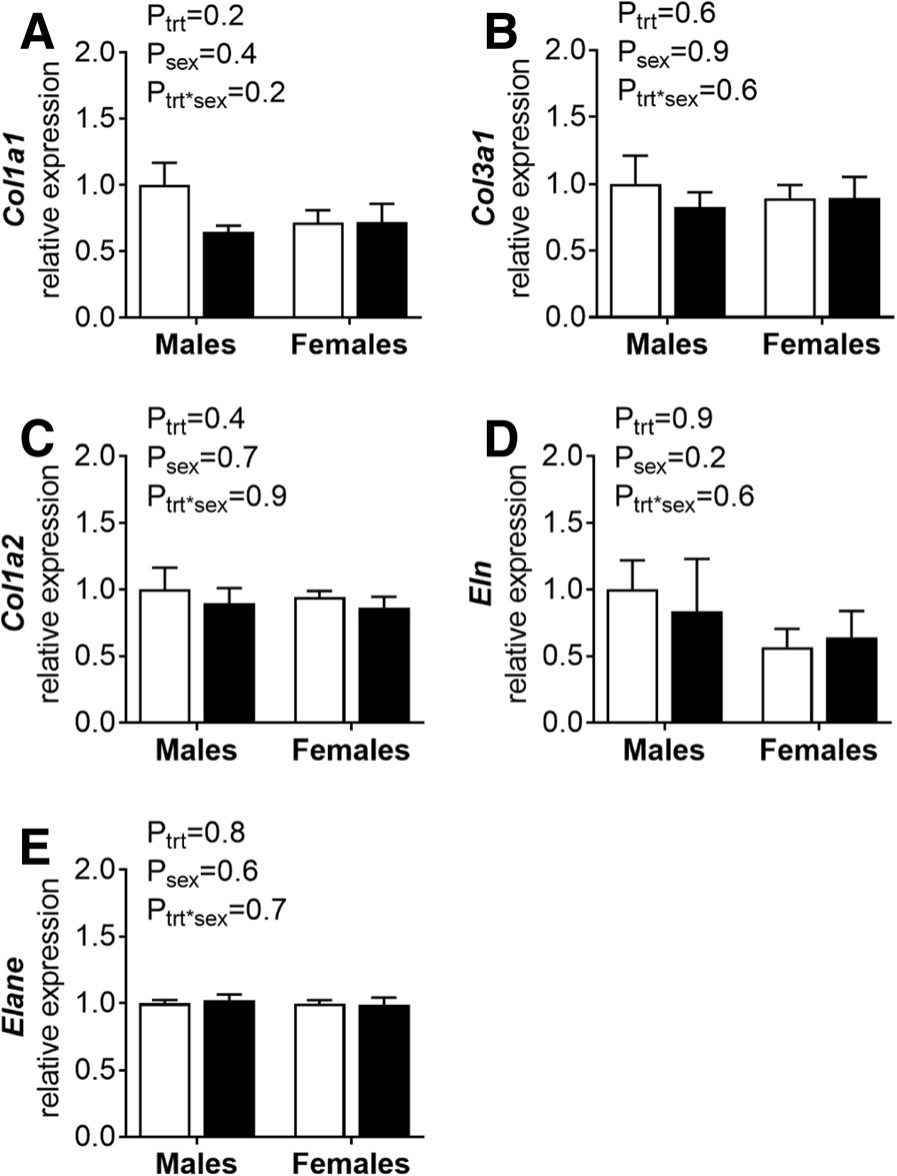
mRNA expression in renal lobar arteries. Quantitative real-time-PCR analysis of renal lobar arteries from male and female offspring at 12 months of age. **a** *Col1a1*, **b** *Col3a1*, **c** *Col1a2*, **d** *Eln* and **e** *Elane* mRNA expression normalised to the mean expression of *18s* and *Hprt.* Control: open points; EtOH-exposed: closed points. Values are mean ± SEM, *n* = 6–8/group. Data analysed using a two-way ANOVA

Relative mRNA expression of *collagen 1a1*, *collagen 3a1*, *collagen 1a2*, *elastase 2* and *elastin* within renal arteries of 12-month-old rats was not altered by EtOH or sex (Fig. 6).

### Offspring biometry at 12 months of age

Body weight and organ weights, absolute or corrected for body weight, were not different between control and EtOH-exposed offspring at 12 months of age (see Additional File 3). Female offspring had lower body, kidney, heart and liver weights compared with male offspring (*P*_sex_ < 0.05; Additional File 3), but this effect was lost when corrected for body weight. Absolute brain weight was not significantly different between groups; however, female offspring had greater brain-to-body weight ratios compared with male offspring, irrespective of prenatal exposure to EtOH (*P*_sex_ < 0.0001; Additional File 3).

## Discussion

Exposure to alcohol throughout gestation affects one in ten pregnancies worldwide [1]. In Western societies, women who drink throughout pregnancy do so at rates of 1–2 standard drinks of alcohol per day [11, 34–36]. Yet, there is limited understanding of the long-term cardiovascular outcomes for the offspring. Here, we report that exposure to chronic low EtOH throughout pregnancy is associated with reduced arterial pressure and an attenuated pressor response to restraint stress in female rat offspring. Cardiovascular changes occurred without the confounding effects of growth restriction or accelerated catch-up growth often observed in more severe models of alcohol exposure [18, 37]. By contrast, male offspring were largely unaffected by ethanol exposure during gestation. Reactivity and passive mechanical properties of renal lobar arteries were unaltered by prenatal ethanol exposure. Together, these data suggest prenatal exposure to alcohol elicits significant changes to the regulation of arterial pressure in a sexually dimorphic manner.

Pronounced hypotension during both active and quiescent phases was observed in female offspring exposed to alcohol throughout pregnancy. Importantly, the lower blood pressure in the EtOH-exposed female offspring was not due to a reduction in locomotor activity, an essential determinant of cardiovascular arousal [38, 39], or alterations in lobar artery reactivity or passive mechanical wall properties. These findings were surprising given our previous study in the same model revealed rats exposed to prenatal alcohol had evidence of cardiovascular dysfunction by 8 months of age, namely reduced maximal aortic flow velocity in both sexes and left ventricular hypertrophy and fibrosis that was greater in female EtOH-exposed offspring compared to males [20]. Our findings of hypotension are in contrast to previous preclinical studies where alcohol exposure in utero was associated with elevated blood pressure in rat offspring, as measured by tail cuff plethysmography (chronic low dose, 6.36% vol/vol ethanol in a liquid diet from E2-birth) [19] and by an indwelling tail-artery catheter (binge high dose model, 1 g/kg ethanol from E13.5-E14.5) [18]. The discrepancy between these studies and our finding may lie in technical differences in the method of blood pressure measurement. Radiotelemetry recording, as used in the current study, is widely recognised as the gold-standard of blood pressure measurements in rodents [40]. Furthermore, Turcotte and colleagues [19] did not specify sex of the offspring studied and therefore it cannot be determined if blood pressure outcomes were sex-specific.

To our knowledge, no clinical study has investigated blood pressure outcomes in human adults exposed to prenatal alcohol. One human study showed blood pressure was normal in 9-year-old children exposed to prenatal alcohol during the first and second trimesters of pregnancy [11]. Functional reserve in the heart, vasculature and the kidneys declines with age, and this effect hastens when compounded by adverse factors such as diabetes, obesity and smoking [41]. Therefore, it is important to determine whether prenatal alcohol exposure affects the rate of cardiovascular ageing in the human population and if cardiovascular sequelae manifest in conditions of stress or disease. The only study to-date in humans to examine long-term cardiovascular complications revealed maternal alcohol exposure was associated with mild chronic kidney disease in offspring at 30 years of age, with the effect greatest in female offspring compared to males [42]; blood pressure was not reported in this study. As CVD and associated kidney injury are most often diseases of late adulthood or the elderly, consideration of older cohorts in both preclinical and clinical studies is warranted. Importantly, the rats examined in this study were over 12 months of age (equivalent to middle age in humans) and therefore it would be of interest to examine if hypotension persists in these animals until late life and whether this is associated with the occurrence of adverse events such as falls or functional decline [43]. Sub-optimal blood pressure levels should be minimised to prevent hypotensive symptoms, particularly in the elderly who are vulnerable to falls [44]. On the other hand, the hypotensive phenotype in female ethanol-exposed offspring may buffer other hallmarks of disease previously reported in these offspring including altered glucose handling [22], and left ventricular hypertrophy and fibrosis [20] and thus could be construed as cardioprotective.

We posed a restraint stress challenge to the offspring to examine whether cardiovascular responsiveness to an environmental stressor could be influenced by prenatal ethanol exposure. Both male and female offspring exposed to alcohol exhibited blunting of the rapid increase in blood pressure observed in control offspring during 30 min of restraint, although this effect was greater in females. The tachycardiac response remained intact in both sexes. Furthermore, staff entering the room in which the rats were housed for scheduled daily inspections led to a peak in arterial pressure, heart rate and locomotor activity in control offspring. This ‘stress’ effect was blunted in ethanol-exposed offspring matching the restraint stress response differences we demonstrated. In humans, heightened cardiovascular reactivity in response to negative stress is considered a risk factor for hypertension and cardiovascular disease [25, 26]. This suggests ethanol-exposed offspring are not at risk of developing hypertension in later life. There has been some research on the association between prenatal ethanol exposure and cardiovascular reactivity in humans; however, these studies were limited to infants. Fifer et al. [45] found infants exposed to prenatal ethanol did not respond to a tilt test, and suggested this altered autonomic cardiac function may increase risk of sudden infant death syndrome. This study is consistent with reduced behavioural arousal observed in infants exposed to prenatal alcohol throughout pregnancy [34, 46]. Oberlander and colleagues (2010) showed that infants exposed to high levels of alcohol during pregnancy had blunted responses to a physical stressor (heel-lance blood draw) and low salivary cortisol levels, which the authors postulated to underlie the altered stress reactivity in these infants. Recently, we have reported that exposure to alcohol in utero in only the periconceptional period (12.5% vol/vol EtOH liquid diet from four days before conception until E4) leads to reduced pressor responsiveness in aged female but not male rat offspring [47]. This was associated with low plasma corticosterone and altered expression of key genes in the stress response pathway in the hippocampus (*Nr3c1*, *Hsp90a1*) and adrenal glands (*Hsp90a1*), suggesting prenatal alcohol exposure programs sex-specific alterations in the hypothalamic-pituitary axis. Blunting of behavioural and physiological responsiveness seen in clinical studies and our animal model may be the result of compensatory or adaptive responses to negative environmental stimuli in utero*.* We have previously shown adult ethanol-exposed male and female offspring exhibited anxiety-like behaviour and presented with altered dendritic morphology within the basolateral amygdala [48]. This suggests that this low level of ethanol exposure in pregnancy can elicit behavioural changes in offspring. Thus, examination of the behavioural responsiveness to an environmental stressor would be worthy of investigation in the future.

Attenuated blood pressure reactivity to stressors may be mediated by multiple central and peripheral mechanisms [49]. Prenatal alcohol exposure has different effects on vascular contraction and endothelial function across the vasculature, with suppression of contraction and enhanced relaxation previously observed in renal lobar arteries from fetal alcohol-exposed sheep [16]. Our renal lobar artery experiments showed α_1_-adrenergic sensitivity, smooth muscle contraction to HiK, perivascular nerve stimulation and passive mechanical wall properties were not affected by prenatal ethanol. These findings are similar to those of Turcotte and colleagues, in which rat offspring exposed to low chronic ethanol (6.36% vol/vol in liquid diet, E2-birth) presented with normal aortic constriction [19]. Thus, these vascular beds are unlikely to be contributing mechanisms to the reduced pressor response in this model. However, we cannot discount the possibility that changes may be observed in other vascular beds, as programming of vascular deficits can be region-specific [16, 50]. We found no differences in renal endothelial or smooth muscle function between males and females. Of particular interest, there are known sex differences in the response of the renal arteries to angiotensin II in young rodents. A previous study showed renal interlobar arteries from 3-month-old mice contracted significantly less to angiotensin II compared to arteries from age-matched male mice, an effect due to angiotensin type 2 receptor-mediated nitric oxide release in females [51]. Key vascular regulatory pathways are balanced towards cardioprotection in females between puberty and menopause, after which the balance shifts to become pro-hypertensive [52]. It is likely the differences between our findings and Viegas et al. [51] is a factor of ageing as the rats used in this study were 12 months of age, considered to be middle age in the Sprague-Dawley where early reproductive senescence may arise [27].

The absence of overt changes in the renal vasculature suggests alterations in the hypothalamic pituitary axis (HPA) and/or brainstem regulation of autonomic and endocrine outputs in the periphery may account for the reduced responsiveness to restraint stress in ethanol-exposed offspring. The fetal HPA axis is highly susceptible to the effects of alcohol exposure in pregnancy [53], with dysregulation evident at multiple levels of the HPA axis in adult rodent and primate offspring [34, 37, 53, 54]. In particular, both hyper- and hypo-responsiveness to aversive stimuli are common outcomes in both preclinical and clinical gestational alcohol studies [53]. In a similar model of chronic alcohol exposure, Lan and colleagues [54] reported that 3-month-old rats exposed to moderate ethanol throughout pregnancy (reaching a BAC of ~ 0.19%) exhibited hyper-responsiveness of the HPA axis in response to a 30-min restraint stress. By contrast, several studies of infants exposed to moderate-to-heavy levels of alcohol in utero demonstrated blunted cardiac responses to a physical stressor [34, 45, 46]. Similarly, we report cardiovascular hypo-responsiveness to stress in adult female EtOH-exposed rat offspring. The reason for these discrepancies between these preclinical rodent studies are unclear, although it is important to note that outcomes appear to be dependent on the timing and level of alcohol exposure (i.e. chronic alcohol intake vs. a ‘binge’ model) [18, 19], the presence of complicating factors such as smoking and drug use [34, 46, 55], the type of stressor, the age and sex of offspring examined in the study and endpoints measured. Prenatal alcohol is also associated with sexually dimorphic effects on the HPA, suggesting a role for sex hormones in mediating the effects of alcohol on HPA activity and regulation. To our knowledge, this is the first study to demonstrate reduced blood pressure reactivity to stress in offspring exposed to low, chronic ethanol throughout pregnancy. Future studies may wish to examine the contributions of the HPA axis to cardiovascular phenotypes in both sexes.

Sex differences in the prevalence of fetal alcohol spectrum disorder (FASD) have been reported in several populations. The incidence of FASD per live birth rate was 1.4 times higher for boys than girls in a Canadian province [56], and a study of 1400 cases of maternal alcoholism in the USA revealed boys had greater alcohol-related cognitive and behavioural dysfunction than girls [57]. By contrast, other epidemiological studies have failed to detect significant sex differences in FASD rates [58, 59]. These discrepancies may be a factor of timing and/or dose of alcohol exposure during pregnancy, which will differentially influence development of particular organ systems including brain, heart, kidney and even the placenta [60]. Numerous experimental studies indicate cardiovascular outcomes may be modulated by both sex and age of offspring. In particular, the neurohumoral regulation of blood pressure may be influenced by sex hormones and sex-specific expression of sex hormone receptors that may alter structure and function of neural systems (for review, see [61]). Furthermore, temporal sex differences in expression of components of the renin-angiotensin system, renal nerve activity, oxidative stress and endothelin influence blood pressure outcomes [23, 27]. Models of developmental programming frequently report elevations in blood pressure in young male offspring, but with young female offspring remaining normotensive. However, cardiovascular risk increases with age in female offspring in association with reproductive senescence and by middle age (12 months of age in rats), female cardioprotection is lost [23, 27]. Therefore, it is possible that sex hormones may contribute to the sexual dichotomy in blood pressure in this model. However, future studies are required to tease out the underlying mechanisms.

## Conclusions

Despite public health efforts, alcohol consumption during pregnancy is common worldwide and confers an increased risk of adult disease. Understanding cardiovascular outcomes of affected adult offspring is of great importance. There are very few preclinical studies in this area of research, and these studies are frequently complicated by additional exposures such as smoking and drug use [11, 34]. Our findings in rat offspring exposed to chronic alcohol during pregnancy are alarming given the absence of overt signs of FASD such as growth restriction and neurological deficits in the present model. Furthermore, this study suggests that the vulnerability of the fetus to the teratogenic effects of alcohol may be influenced by the sex of the fetus. Our studies confirm that abstinence from alcohol is the safest option during pregnancy, as recommended by public health organisations in Australia, the USA, Canada, Denmark, France and the World Health Organisation. Decreasing or eliminating alcohol use during pregnancy may reduce the severity of cardiovascular dysfunction in offspring.

## Additional files

Additional file 1: **Table S1. **Primer sequences for quantitative real-time PCR. (DOCX 13 kb)

Additional file 2: Renal artery contraction evoked by perivascular nerve stimulation of increasing voltage and frequency. Constrictions evoked by increasing stimulus strength and increasing stimulus frequency in isolated renal lobar arteries from offspring at 12 months of age. Dashed lines represent the responses in the presence of α_1_-adrenoreceptor antagonist prazosin (10^− 6^ M). Dotted lines represent the responses in the presence of voltage-dependent Na^+^ channel blocker tetrodotoxin (TTX, 10^− 7^ M). Control: open points; EtOH-exposed: closed points. Values are mean ± SEM, *n* = 7–9/group. Data analysed using repeated-measures two-way ANOVA; (A) P_trt_ = 0.02, (B-K). No significant differences between treatment groups were observed. (DOCX 298 kb)

Additional file 3: Body weight and organ weights at 12 months of age. Values are mean ± SEM; *n* = 6–7 per group. All data analysed by two-way ANOVA. NS, not significant. (DOCX 16 kb)
