# Supplementary material for: Chronic low alcohol intake during pregnancy programs sex-specific cardiovascular deficits in rats
Source: Biol Sex Differ. 2019 Apr 22;10:21. doi: 10.1186/s13293-019-0235-9 (PMC6477739; doi:10.1186/s13293-019-0235-9)
Supplement: Supplementary file 1 — Table S1. Primer sequences for quantitative real-time PCR. (DOCX 13 kb) [file 13293_2019_235_MOESM1_ESM.docx]

Table 1. Primer sequences for quantitative real-time PCR.

| **Gene** | **Forward primer (5’ to 3’)** | **Reverse primer (5’ to 3’)** |
| --- | --- | --- |
| *Col1a1* | TGGATTCCAGTTCGAGTATG | AGTGATAGGTGATGTTCTGG |
| *Col1a2* | AGTGGAAGAGCGATTACTAC | ATTGATGGTCTCTCCTAACC |
| *Col3a1* | TTTCAAGATCAACACTGAGG | TATTCTCCGCTCTTGAGTTC |
| *Elastase 2* | CACTCGACAAATCTTCTCTG | TGAGCTGGATAATCACAATG |
| *Elastin* | AAACCTTCTATGACCACCC | CTGCTGTCTGATTTCCTTG |
| *18s* | TTATCTAGAGTCACCAAGCC | CAGTTATGGTTCCTTTGGTC |
| *Hprt* | ACTGGTAAAACAATGCAGAC | CCTGAAGTGCTCATTATAGTC |
