# Supplementary figures and images for: Chronic low alcohol intake during pregnancy programs sex-specific cardiovascular deficits in rats
Source: Biol Sex Differ. 2019 Apr 22;10:21. doi: 10.1186/s13293-019-0235-9 (PMC6477739; doi:10.1186/s13293-019-0235-9)

**
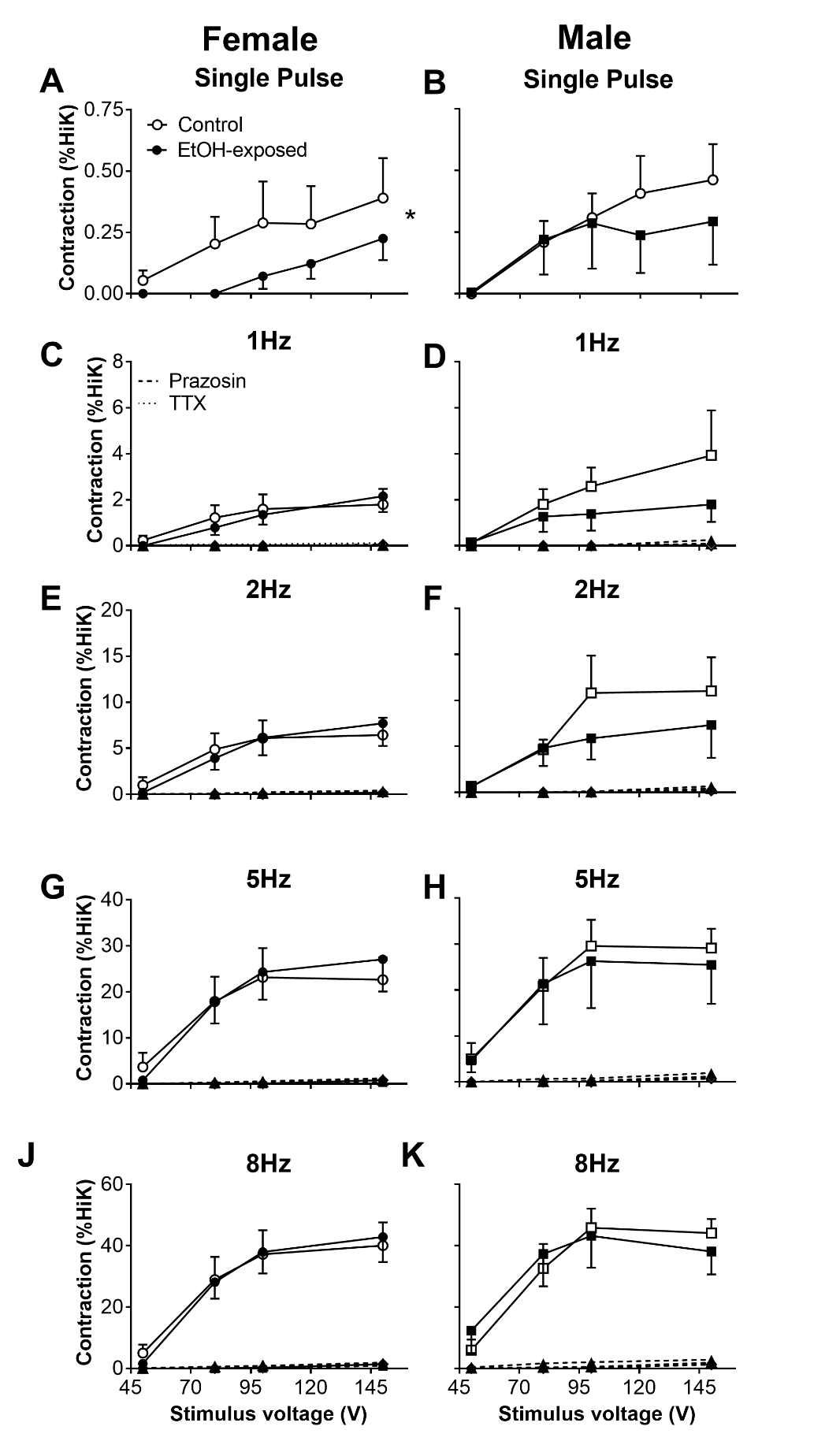
**

Supplement: Supplementary file 2 — Renal artery contraction evoked by perivascular nerve stimulation of increasing voltage and frequency. Constrictions evoked by increasing stimulus strength and increasing stimulus frequency in isolated renal lobar arteries from offspring at 12 months of age. Dashed lines represent the responses in the presence of α1-adrenoreceptor antagonist prazosin (10− 6 M). Dotted lines represent the responses in the presence of voltage-dependent Na+ channel blocker tetrodotoxin (TTX, 10− 7 M). Control: open points; EtOH-exposed: closed points. Values are mean ± SEM, n = 7–9/group. Data analysed using repeated-measures two-way ANOVA; (A) Ptrt = 0.02, (B-K). No significant differences between treatment groups were observed. (DOCX 298 kb) [file 13293_2019_235_MOESM2_ESM.docx]
