# Supplementary material for: Chronic low alcohol intake during pregnancy programs sex-specific cardiovascular deficits in rats
Source: Biol Sex Differ. 2019 Apr 22;10:21. doi: 10.1186/s13293-019-0235-9 (PMC6477739; doi:10.1186/s13293-019-0235-9)
Supplement: Supplementary file 3 — Body weight and organ weights at 12 months of age. Values are mean ± SEM; n = 6–7 per group. All data analysed by two-way ANOVA. NS, not significant. (DOCX 16 kb) [file 13293_2019_235_MOESM3_ESM.docx]

**Additional file 3.** Body weight and organ weights at 12 months of age.

|  | Females | | Males | | Two-way ANOVA | | |
| --- | --- | --- | --- | --- | --- | --- | --- |
|  | Control | EtOH-exposed | Control | EtOH-exposed | P_trt_ | P_sex_ | P_trt*sex_ |
| BW (g) | 356 ± 9 | 333 ± 13 | 548 ± 17 | 559 ± 14 | NS | P<0.0001 | NS |
| Kidney weight (g) | 1.56 ± 0.09 | 1.17 ± 0.12 | 2.26 ± 0.19 | 2.33 ± 0.05 | NS | P<0.0001 | NS |
| Kidney:BW (x10^-3^) | 4.40 ± 0.28 | 3.58 ± 0.26 | 4.11 ± 0.26 | 4.12 ± 0.11 | NS | NS | NS |
| Heart weight (g) | 1.68 ± 0.11 | 1.32 ± 0.14 | 2.13 ± 0.15 | 2.03 ± 0.16 | NS | P=0.0006 | NS |
| Heart:BW (x10^-3^) | 4.74 ± 0.31 | 3.96 ± 0.34 | 3.90 ± 0.25 | 3.63 ± 0.27 | NS | NS | NS |
| Liver weight (g) | 15.27 ± 0.58 | 13.73 ± 1.26 | 22.97 ± 2.33 | 24.79 ± 1.69 | NS | P<0.0001 | NS |
| Liver:BW (x10^-3^) | 43.03 ± 4.20 | 41.50 ± 3.47 | 41.55 ± 2.83 | 44.26 ± 6.65 | NS | NS | NS |
| Brain weight (g) | 2.41 ± 0.08 | 2.52 ± 0.08 | 2.57 ± 0.11 | 2.53 ± 0.08 | NS | NS | NS |
| Brain:BW (x10^-3^) | 6.84 ± 0.36 | 7.59 ± 0.20 | 4.73 ± 0.28 | 4.55 ± 0.20 | NS | P<0.0001 | NS |

Values are mean ± SEM; *n*=6-7 per group. All data analysed by two-way ANOVA. NS, not significant.
